# Freshwater Environmental Risk Assessment of Down‐the‐Drain Octinoxate Emissions in the United States

**DOI:** 10.1002/etc.5488

**Published:** 2022-10-25

**Authors:** Emily E. Burns, Kyle S. Roush, Susan A. Csiszar, Iain A. Davies

**Affiliations:** ^1^ Personal Care Products Council Washington District of Columbia USA; ^2^ Procter & Gamble Cincinnati Ohio USA

**Keywords:** 2‐Ethylhexyl‐4‐methoxycinnamate, EHMC, environmental modeling, exposure modeling, personal care products, risk assessment, UV filter

## Abstract

Organic ultraviolet (UV) filters are used in a variety of cosmetic and personal care products (CPCPs), including sunscreens, due to their ability to absorb solar radiation. These UV filters can be washed down the drain through bathing, cleansing, or the laundering of clothing, therefore UV filters can enter the freshwater environment via wastewater treatment plant effluent, and so a freshwater risk assessment is necessary to establish the environmentally safe use of these important CPCP ingredients. In the present study, an environmental safety assessment for a UV filter of regulatory concern, octinoxate, was conducted. An established risk assessment framework designed specifically for CPCPs released to the freshwater environment in the United States was used for the assessment. A distribution of predicted environmental concentrations (PECs) representative of conditions across the region was calculated using the spatially resolved probabilistic exposure model iSTREEM. A review of available hazard data was conducted to derive a predicted no‐effect concentration (PNEC). The safety assessment was conducted by comparing the PEC distribution to the PNEC. A substantial margin of safety was found between the 90th percentile PEC, which is representative of the reasonable worst‐case environmental exposure, and the PNEC. Owing to this finding of negligible risk, further refinement of the risk assessment through the generation of experimental data or refinement of conservative assumptions is not prioritized. These results are critical for demonstrating the environmental safety of UV filters in the US freshwater environment and will help guide future work. *Environ Toxicol Chem* 2022;41:3116–3124. © 2022 The Authors. *Environmental Toxicology and Chemistry* published by Wiley Periodicals LLC on behalf of SETAC.

## INTRODUCTION

Organic ultraviolet (UV) filters are important ingredients in a variety of cosmetic and personal care products (CPCPs), most notably sunscreens, due to their ability to absorb UV rays (Manová et al., 2013). By reducing exposure to UV radiation, UV filters can play an important role in preventing skin cancers (e.g., squamous cell carcinomas and melanoma), sunburn, and photoaging, thereby making them an important component of sun safety practice (Marson et al., 2021; Sander et al., 2020). Although there are significant benefits to the use of UV filters on human health, the environmental impact of many UV filters is under investigation. Most recently, the United States Food and Drug Administration issued their intent to publish an Environmental Impact Statement for two important UV filters, oxybenzone (BP‐3) and octinoxate (EHMC). A robust environmental risk assessment (ERA) for BP‐3 in US freshwaters was recently published (Burns et al., 2021), and the present study presents a comparable assessment for the other UV filter of regulatory interest, EHMC.

Although there has been recent scientific interest in the direct wash‐off of UV filters during aquatic recreation and the subsequent potential impact of this exposure on marine ecosystems (Mitchelmore et al., 2021; Watkins & Sallach, 2021), EHMC is also expected to enter the freshwater environment through the down‐the‐drain wash‐off of CPCPs, such as daily use products and including sunscreens. Cosmetic and personal care products are released down the drain through the washing, rinsing, or cleansing of skin or clothing. Once released to wastewater, UV filters will be subject to wastewater treatment and the remaining fraction released to the freshwater environment through wastewater effluent. Monitoring data from wastewater treatment plant (WWTP) effluent and receiving waters confirm that this exposure pathway is relevant for octinoxate (Ekpeghere et al., 2016; Hu et al., 2021; Wright, 2021), therefore it is sensible to conduct a down‐the‐drain US freshwater ERA for EHMC.

Risk assessment is critical to ensuring the environmentally safe use of consumer chemicals, such as CPCPs. Standardized ERA methods are available, and an approach based on these methods has been applied previously for UV filters (Burns et al., 2021) and other ingredients with down‐the‐drain environmental releases (Cowan‐Ellsberry et al., 2014; McDonough et al., 2017). In short, environmental exposure is characterized as a predicted environmental concentration (PEC) and compared with the environmental hazard potential represented as a predicted no‐effect concentration (PNEC). The assessment follows a tiered approach where lower tiers incorporate conservative assumptions and available data, and if the PEC is less than the PNEC, this strongly suggests the chemical is unlikely to pose an unacceptable risk to the environment (Nabholz, 1991). At lower tiers, if the PEC is equal to or greater than the PNEC, then higher‐tier refinements can be made, potentially through the collection of additional data or refinement of conservative assumptions, prior to concluding on risk (Salvito et al., 2002; US Environmental Protection Agency [USEPA], 1998).

A literature review of EHMC freshwater monitoring data identified no studies conducted in the United States, similar to the review for BP‐3 (Burns et al., 2021). Due to the lack of monitoring data specific to the United States, a robust exposure modeling approach is needed to predict concentrations from down‐the‐drain wash‐off of EHMC that reflect the variability in population and geographical features across the region. When monitoring data are not available, the use of exposure models to derive PECs is standard proactive practice, including regulatory ERA frameworks in the United States and the European Union (European Commission Joint Research Centre [EC JRC], 2003; Zeeman & Gilford, 1993). For a US‐wide exposure assessment, the iSTREEM model is a robust and well‐established approach for consumer chemicals released down the drain (Aronson et al., 2012). The iSTREEM model is a spatially explicit exposure model which provides aquatic exposure distribution predictions representative of the conterminous United States (Kapo et al., 2016). The predictions from iSTREEM have been demonstrated to be conservative, yet realistic, for consumer product ingredients (Kapo et al., 2016; Sanderson et al., 2013), including the UV filter BP‐3 (Burns et al., 2021).

The objective of the present study was to provide a novel ERA of EHMC emissions in the United States given substantial known use and significant scientific interest. Best available data were utilized to conduct a high‐tier assessment that evaluated both the ecological safety of the material of concern and the utility of robust predictive tools within the risk assessment framework. Specifically, EHMC hazard data were reviewed and a US‐wide exposure modeling assessment of EHMC from CPCP emissions was conducted. The results were used to perform a risk characterization through comparison of the PEC distribution with the derived PNEC. The present study built on a previously reported framework for down‐the‐drain risk assessment of CPCP ingredients in the United States (Burns et al., 2021).

## METHODS

The present study provides an assessment of the environmental safety of EHMC released down the drain from CPCPs, which includes sunscreens, in the United States. These emissions flow predominantly to WWTPs and are released as treated effluent to the freshwater environment, therefore environmental safety was assessed in receiving rivers. The ERA follows the methods previously published in a similar nationwide down‐the‐drain assessment of BP‐3 (Burns et al., 2021) and consists of a review of key chemical properties, exposure assessment, hazard assessment, and risk characterization.

### Chemical properties

Considering the environmental compartment being assessed (freshwater) and the modeling approach applied in the present study, the key physicochemical properties required for EHMC (CAS RN 5466‐77‐3, 83834‐59‐7) are described in Supporting Information, Table S1. A screening biodegradability test for EHMC demonstrated that it is readily biodegradable because it reached 78% biodegradation in 28 days and passed the 10‐day window in a manometric respirometry test (Organisation for Economic Co‐operation and Development [OECD] test guideline 301F), retrieved from the European Chemicals Agency (ECHA) registration dossier (ECHA, 2012).

### Exposure characterization

Exposure was characterized using EHMC emissions from CPCPs (including sunscreens) and the freely available web‐based US exposure model, iSTREEM. Briefly, iSTREEM is a digitized river network representative of the United States and comprises 227 876 unique river segments for which a chemical concentration is estimated for each segment. Within each segment, chemical inputs include upstream contributions and WWTP discharges (if applicable) and losses due to in‐stream decay (biodegradation). The result is a distribution of surface water concentrations representative of the nation‐scale variability in chemical inputs and river flows.

The annual usage of EHMC in CPCPs was estimated using 2019 United States market sales data from Euromonitor International (Euromonitor, 2021). It was assumed that 100% of this usage would be released down the drain, which is a conservative estimate (Burns et al., 2021), but a suitable assumption for an initial safety assessment. The Euromonitor EHMC volume data were converted to the iSTREEM compatible emission, grams/per capita/per day (g/c/d), resulting in a final EHMC emission of 0.0086 g/c/d.

The WWTP removal value used in the modeling was 83.5%, the average of 24 removal values reported in the literature (see Supporting Information, Table S2). Removals from WWTPs that use activated sludge treatment were collected from the literature. The mean measured WWTP removal was leveraged for all iSTREEM biological treatment input types, oxidation ditch, lagoon, trickling filter, and rotating biological contractor, whereas primary removal was set to zero (Burns et al., 2021), the latter of which is a conservative assumption. In‐stream decay was characterized by a generic first‐order rate constant (*k*) assigned according to the result of the biodegradation screening test (i.e., readily biodegradable, passing the 10‐day window), in this case, 0.047 day‐1 (15‐day half‐life; EC JRC, 2003). A summary of iSTREEM inputs can be found in Supporting Information, Table S3.

### Hazard characterization

Briefly, publicly available environmental toxicity data for EHMC were collected and screened for effects on ecologically relevant endpoints (e.g., mortality, growth, reproduction) as a result of aqueous exposure. Studies conducted according to or similar to standardized test guidelines were considered most reliable and relevant. Octinoxate freshwater hazard was characterized via a PNEC, calculated by division of a selected ecotoxicity value (representative of a sensitive ecological endpoint) by a suitable application factor, a defined uncertainty factor that conservatively accounts for variation in chemical sensitivity, exposure length, data availability, and so on. The EnviroTox PNEC Calculator Tool, which considers breadth of test species data, test type (i.e., acute or chronic), and region of interest (i.e., United States), was used to select an appropriate and localized application factor (Supporting Information, Table S4) that aligns with the regional regulatory authority (i.e., US Environmental Protection Agency [USEPA]) PNEC guidance (Health and Environmental Sciences Institute, 2022).

### Risk characterization

The safety assessment was conducted by comparing PEC percentiles (25th, 50th, 75th, and 90th) to the PNEC. Comparison with the 50th percentile is ideal because this is the midpoint of the dataset (*n* = 227 846) where the data are robust and not skewed by outliers produced by the model (Kapo et al., 2016). Comparison with the 90th percentile is consistent with regulatory ERA guidance (EC JRC, 2003; Nabholz, 1991) and represents a “reasonable worst‐case” PEC (Burns et al., 2021). The environmental risk is considered low or negligible if the PEC is less than the PNEC (ECHA, 2012). The ERA approach is summarized in Supporting Information, Figure S1.

## RESULTS AND DISCUSSION

### PECs of EHMC

**Figure 1:**
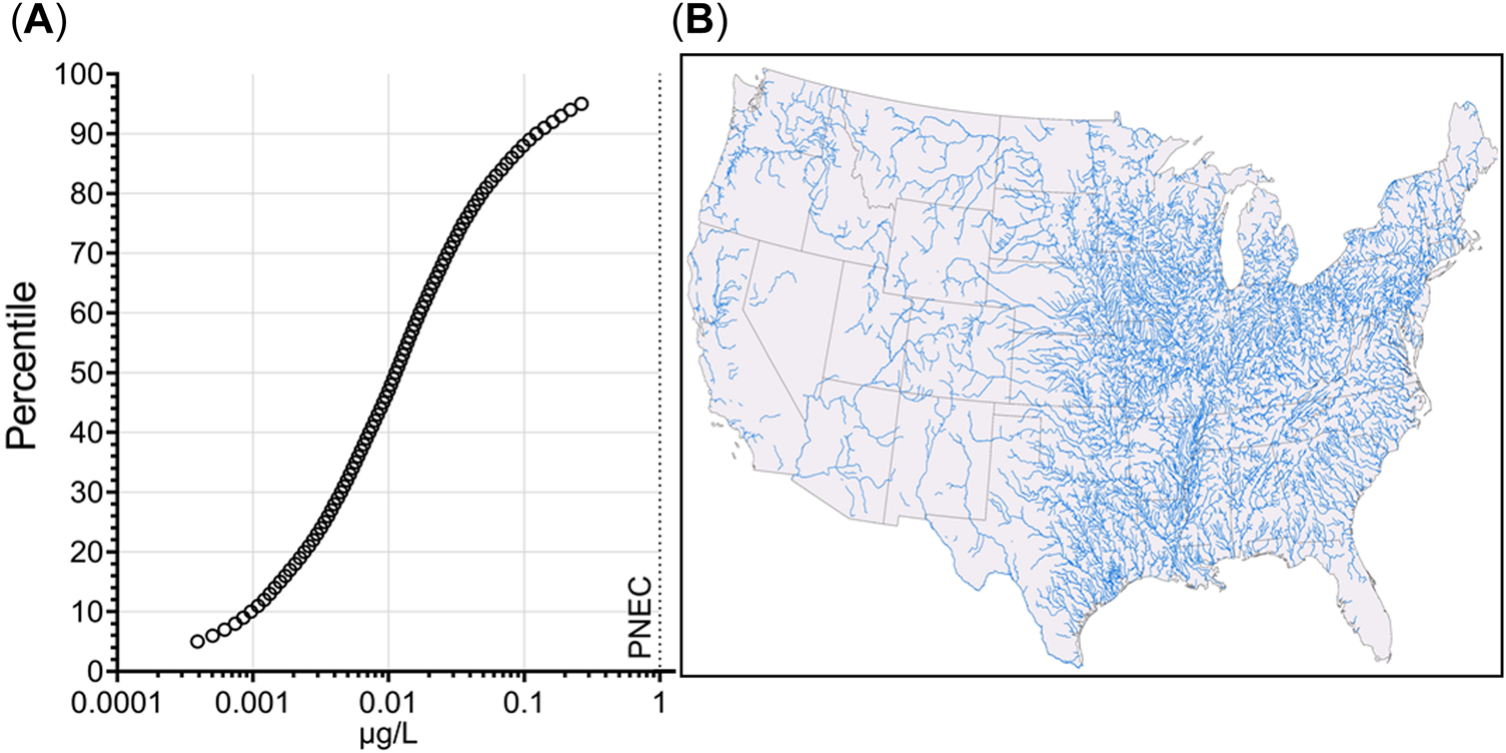
(**A**) Distribution (5th–95th percentile) of modeled octinoxate concentrations per river segment (*n* = 227 876). The predicted no‐effect concentration (PNEC; 1 µg/L) is plotted as a vertical dashed line for comparison. (**B**) Map of the river segments modeled across the United States; this map indicates the spatial extent of the rivers modeled and does not represent actual modeled concentrations.

The distribution of the 5th to 95th percentile predicted mean‐flow concentrations of EHMC released down the drain to rivers in the United States is presented in Figure 1. The median of the predictions (50th percentile) was 0.01 µg/L, indicating predicted concentrations were generally near trace levels. Each modeled segment was representative of the spatial variability in flow combined with the US population, WWTP size and location, and concentrations of EHMC transported from upstream in the conterminous United States.

The PECs were determined using the same conservative approach utilized previously for estimating UV filter emissions to US freshwaters (Burns et al., 2021). For EHMC emissions, the estimate from 2019 rather than 2020 was selected because it was higher (Euromonitor, 2021) and assumed that 100% of this emission will go down the drain. The assumption that 100% of emissions go down the drain was conservative for several reasons. First, Straub (2002) estimated that approximately 10% of a sunscreen product will remain in the packaging and therefore likely go to solid waste. Second, not all EHMC use is in down‐the‐drain applications, for example sunscreens that are used during swimming at lakes and beaches (Balmer et al., 2005; Mitchelmore et al., 2019; O'Malley et al., 2021), which were out of scope in this assessment, but are briefly addressed in the present study.

Beyond emissions, other conservative assumptions which affect the PEC distribution were also made. The average WWTP removal calculated from the 24 values reported in the literature was 83.5%. This estimate was applied to all WWTPs with a biological treatment component, although no removal was assumed in facilities with only primary removal. The SimpleTreat 4.0 model, which predicts removals in an activated sludge treatment plant, predicted an overall removal of 96.4% for EHMC (Struijs, 2014). This provides another line of evidence that the WWTP removal calculated was reasonable. Indeed 50% of the removal values (12 of 24 removal values) from the literature were above the SimpleTreat prediction, which suggests that EHMC is very well removed in activated sludge treatment. Assumptions regarding EHMC decay rate were also conservative. The estimate of in‐stream biodegradation was likely an underestimate because it is based on a method that leverages the results of already highly conservative biodegradation screening tests (Burns et al., 2021). Although biodegradation was expected to be the dominant in‐stream removal mechanism for EHMC, other fate processes could also be impacting the environmental half‐life of EHMC, such as photodegradation (MacManus‐Spencer et al., 2011; Rodil et al., 2009). At this time, only biodegradation was included as an in‐stream attenuation process. Incorporating further environmental fate processes could be investigated along with the refinement of any of the aforementioned conservative assumptions applied if higher‐tier exposure estimates were needed.

Although emissions data were provided on an annual basis, it is conceivable that there will be some seasonal variability in UV filter down‐the‐drain emissions, as discussed in Burns et al. (2021). However, currently available monitoring data suggests that seasonal differences in WWTP effluents and effluent‐receiving rivers are not pronounced. For example, Li et al. (2022) did observe higher concentrations of EHMC in WWTP influent in the warm season compared with the cold season, but this difference was not found to be statistically significant. On the other hand, Tsui et al. (2014) found a 21% difference in EHMC concentrations in wet season versus dry season influent, but differences in effluent were reduced (14%) and not statistically significant. Similarly, Ekpeghere et al. (2016) found an increased UV filter influent load of 27% in the spring versus the fall; however, a consistent seasonal trend was not observed in receiving waters. Overall, present experimental data suggest that differences in seasonal loadings of EHMC are possible (i.e., <30%), but this increase in WWTP influent has not been found to translate into higher concentrations in receiving waters. These findings highlight the complexity of factors that influence environmental concentrations beyond emissions alone.

#### Comparison of predicted concentrations with monitoring data

**Figure 2:**
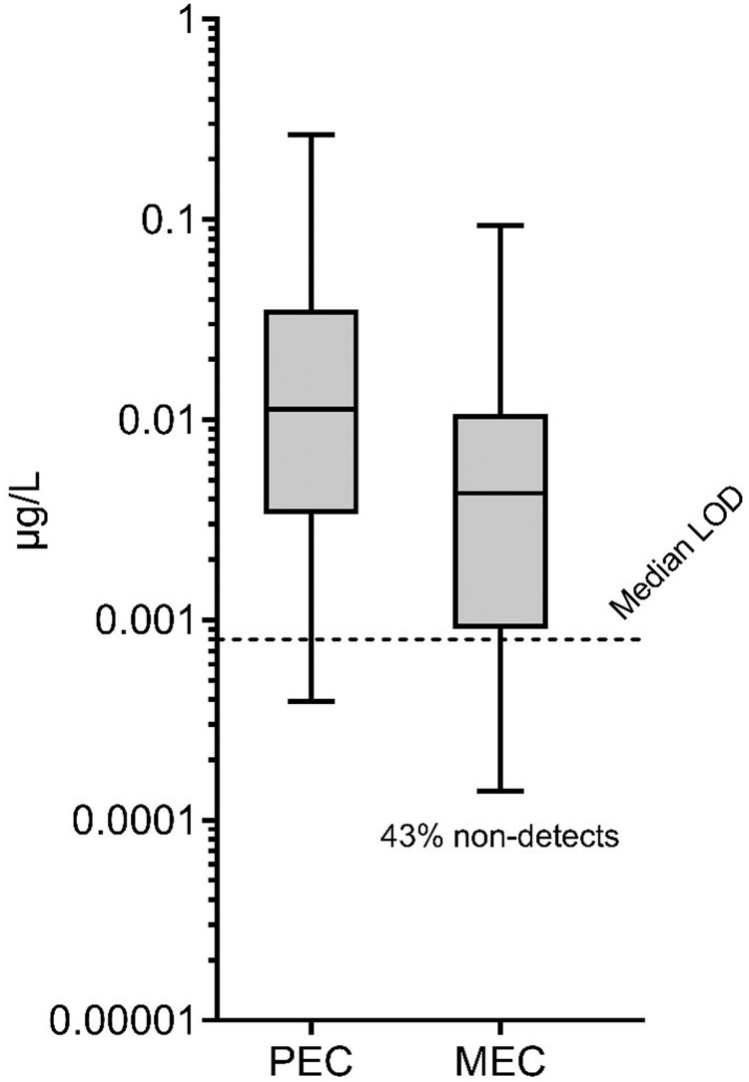
Box plots (5th–95th percentile) of US predicted environmental concentrations (PECs; *n* = 227 876) and global surface water octinoxate concentrations (*n* = 292). The dotted line represents the median limit of detection (LOD) across the monitoring studies surveyed. The top and bottom caps represent the 5th and 95th percentiles, respectively. The line within the boxes represents the median. The bottom and top of the boxes represent the 25th and 75th percentiles, respectively. MEC, measured environmental concentration.

The distribution of EHMC PECs was compared with a global distribution of measured environmental concentrations (MECs) from monitoring studies representative of the modeled environmental exposure scenario (see Supporting Information, Table S5) in Figure 2. A comparison with monitoring data from the United States, while preferable, was not possible because no freshwater measurements were available at the time of writing the present study. Therefore, although not an exact comparison, the global MECs can still provide some insight into whether the PECs were conservative and reasonable.

The global 50th and 90th percentile MECs were 0.004 and 0.04 µg/L, respectively. These values were lower than the predicted 50th and 90th percentiles, 0.01 and 0.12 µg/L, respectively. Importantly, the PEC concentration distribution (e.g., 25th, 50th, 75th, 95th percentiles) all exceeded the MEC distribution, suggesting modeled concentrations were predictive yet conservative. In the MEC dataset, 43% of the values were at or below the limit of detection (LOD). The median LOD across the studies was 0.0008 µg/L, indicating that, in general, the analytical methods used to quantify EHMC in the environment were sensitive and therefore the concentrations of EHMC were detected down to or below trace levels. The global MECs are influenced by a variety of different environmental and socioeconomic factors (Kilgallon et al., 2017) that, together with the sampling strategy (Johnson et al., 2008), will affect the observed results. It was expected that there will be some overlap with US conditions from this dataset because the global data represent similar consumer uses and exposure scenarios in regions where UV filters are used, but the lower concentrations observed could either be the result of differences in per capita usage in combination with differing environmental conditions (e.g., river flow) or a demonstration that the assumptions used for PECs were highly conservative, or both. Furthermore, the MEC distribution presented in the present assessment was conservative due to the data‐handling approach used for data reported as at or below the LOD. All at or below the LOD values were censored with their corresponding LOD, when in reality the true value could be anywhere between zero and the LOD. Although there are other censoring approaches available (e.g., George et al., 2021), the goal of this exercise was risk assessment, thus the most conservative approach to incorporating values below the LOD was selected. The MEC comparison was not meant to corroborate modeling output, but rather function as a secondary line of evidence that PECs were protective, yet reasonable (Burns et al., 2021). Taken together, these results strongly suggest that modeled concentrations were reasonable and conservative, and therefore suitable for a US risk assessment.

There was a single influent concentration from the United States available to compare with our predicted WWTP loadings. Loraine and Pettigrove (2006) reported a WWTP influent concentration of 0.4 µg/L in a southern California treatment facility during the wet season. Interestingly, the sampled EHMC influent concentration was below the LOD (0.28 µg/L) when the WWTP was surveyed in the dry season. These influent concentrations were near the first percentile influent PEC, which was similar to how concentrations for BP‐3 from this same monitoring study compared with influent PECs following the same exposure assessment framework (Burns et al., 2021). Although this comparison was limited, it does provide some evidence that the predicted down‐the‐drain release of EHMC to WWTP influent was highly conservative.

Although the comparisons with MECs presented in our risk assessment had some limitations, they served as an additional line of evidence that the PECs for EHMC were conservative and thus suitable for a freshwater risk assessment of EHMC released down the drain. These results followed closely the outcome for BP‐3 (Burns et al., 2021), which suggests the exposure assessments for UV filters conducted within this iSTREEM framework are protective for the US freshwater environment and therefore suitable for risk assessment.

### Hazard characterization

Aquatic toxicity data for EHMC exposure were available across multiple trophic levels, including those of primary interest in the aquatic risk assessment framework—algae, invertebrates, and fish. It is important to note that many of the aqueous EHMC were conducted at concentrations above water solubility, which has been measured as 51 µg/L (ECHA, 2012). Whether using a solvent or testing with the material undissolved in the test medium, effects seen at test concentrations above the limit of solubility were not representative of the bioavailable fraction and true aquatic toxicity. As such, effects seen below the solubility limit were considered relevant for the ERA. A summary of the most sensitive aquatic toxicity data across trophic levels is found in Table 1.

**Table 1:** Summary of the most sensitive acute and chronic aquatic (freshwater) toxicity data by trophic level used in the predicted no‐effect concentration derivation

Test type	Trophic level	Concentration (µg/L)	Endpoint	Method	Species	Study year	Reference
Acute	Algae	>51^a^	72 h EC50	OECD 201	*Raphidocelis subcapitata*	2000	ECHA (2022)
	Invertebrate	>51^a^	48 h EC50	OECD 202	*Daphnia magna*	2003	ECHA (2022)
	Fish	>51^a^	96 h LC50	OECD 203	*Cyprinus carpio*	2000	ECHA (2022)
Chronic	Algae	>51^a^	72 h NOEC	OECD 201	*R. subcapitata*	2000	ECHA (2022)
	Invertebrate	40	21 days NOEC	OECD 211	*D. magna*	2011	Sieratowicz et al. (2011)
	Fish	**10**	40 days NOEC	Undefined	*Danio rerio*	2019	Zhou et al. (2019)
Assessment factor		10	—	—	—	—	—
Freshwater PNEC		1 µg/L	—	—	—	—	—

^a^ 51 µg/L is the limit of environmental relevance for aquatic exposures (i.e., water solubility).

Other relevant studies referenced in text. Critical toxicity value for predicted no‐effect concentration derivation indicated in bold.

EC50 = median effect concentration; LC50 = median lethal concentration; NOEC = no observed effect concentration; PNEC = predicted no‐effect concentration.

Results from several acute studies of EHMC indicated a lack of significant aquatic toxicity at or below solubility (51 µg/L) across all trophic levels of interest (Cahova et al., 2021; ECHA, 2022; Fent et al., 2010; Jang et al., 2016; Molins‐Delgado et al., 2016; Nataraj et al., 2020; Park et al., 2017; Sieratowicz et al., 2011). Thus, acute aquatic toxicity at environmentally relevant concentrations was not of concern. With regard to chronic exposures, the algae and invertebrate studies also lacked supportable evidence of toxicity at or below the solubility limit of 51 µg/L (ECHA, 2012; Pablos et al., 2015; Sieratowicz et al., 2011). Sieratowicz et al. (2011) was able to derive a chronic invertebrate no observed effect concentration (NOEC) of 40 µg/L (within the soluble concentration range) from a *Daphnia magna* reproduction test (OECD test guideline 211 [2018]; Table 1), but the study was challenged by a lowest observed effect concentration (LOEC) that was above the solubility limit and potential interference by the solvent, which was dosed above the OECD recommended limit (OECD, 2019). The OECD 211 (2018) study from the ECHA dossier conducted at the solubility limit without a solvent indicated no toxic effects to *D. magna*. Alternatively, fish appeared to have some sensitivity to EHMC in chronic studies at or below the solubility limit. A *Danio rerio* fish sexual development test (OECD test guideline 234 [2011]), conducted as part of ECHA regulatory chemical registration, detected effects on growth in the single test concentration conducted at the solubility limit (i.e., ~50 µg/L; ECHA, 2012). Lee et al. (2019) also noted chronic reproductive effects after exposure to 50 µg/L EHMC in a life‐cycle test of *Oryzias latipes*. However, a relevant statistical endpoint for hazard characterization (e.g., NOEC) could not be calculated in either study due to test design (i.e., no concentrations were tested below 50 µg/L). Zhou et al. (2019) was able to derive a chronic fish NOEC of 10 µg/L from a life‐cycle exposure of *D. rerio*. The NOEC was within a soluble concentration range (Table 1), although the study could be refined because the LOEC identified was above the solubility limit.

The freshwater PNEC calculated for EHMC was 1 µg/L (Table 2) and derived as follows. The critical toxicity value was selected from the most sensitive and relevant chronic toxicity endpoints (Table 1). Because effects above water solubility were not considered relevant for the aquatic environment, only NOECs detected below the solubility limit were targeted. Importantly, the primary endpoint of interest, a NOEC of 10 µg/L (Zhou et al., 2019), was considered to be a conservative estimate of toxicity. The wide test concentration gaps (i.e., 10×) utilized in the present study were above test guideline recommendations and led to a lack of granularity on potential effects (or lack thereof) between 10 and approximately 50 µg/L. In the absence of refined test data, the NOEC value of 10 µg/L was used, although the true NOEC may fall closer to the solubility limit. The selected application factor of 10 was considered appropriately protective of the environment and aligned with regulatory guidance given the full suite of acute and chronic toxicity data available. Thus, combining the NOEC of 10 µg/L and an application factor of 10 yielded a PNEC of 1 µg/L.

**Table 2:** Summary of iSTREEM percentiles for 2‐ethylhexyl‐4‐methoxycinnamate exposure in effluent‐receiving rivers in the United States

PEC percentile (µg/L)				PNEC (µg/L)
25th	50th	75th	90th	1
0.003	0.01	0.04	0.1	

PEC = predicted environmental concentration; PNEC = predicted no‐effect concentration.

### Environmental risk characterization

A summary of key EHMC PECs for US risk assessment are presented in Table 2, along with the other key risk assessment metric, the PNEC. The median predicted concentration (50th percentile) was two orders of magnitude less than the PNEC. A similar conclusion can be drawn for the 75th percentile PEC, which indicated that three‐quarters of the PEC dataset was equal to or below 0.04 µg/L, again 25 times less than the PNEC. The 90th percentile PEC was closer to the PNEC, which was expected because this concentration was considered a “reasonable worst‐case” PEC because it represents a concentration well into the right tail of the PEC distribution (McDonough et al., 2016). Despite this, there was still a substantial margin of safety between the 90th percentile PEC and the PNEC. In addition, the 90th percentile PEC was three times larger than the 90th percentile of global MECs (see Figure 2), providing further indication that the US PEC presented was conservative and the risk assessment conclusions were supported. In the highly conservative situation where EHMC loadings were to occur in just half the year (e.g., all EHMC emissions in occurred in warmer months), there was still a significant margin of safety between the 90th percentile PEC (0.24 µg/L) and PNEC (i.e., PEC/PNEC < 0.25). Although the original assumptions made for the PEC were expected to cover temporal variability in emissions (see *PECs of EHMC*), this comparison was conducted as a secondary check for the environmentally safe use of EHMC released down the drain. It is therefore concluded that adverse environmental effects in the freshwater environment due to the down‐the‐drain release of EHMC are considered unlikely. Owing to this finding of negligible risk, further assessment through the collection of higher‐tier environmental fate and toxicity data is not considered a high priority for EHMC in WWTP effluent‐receiving freshwaters of the United States.

A literature search for existing freshwater EHMC ERAs was conducted, and it was determined that the conclusions from those assessments were similar to what was found in this assessment for the United Sates. Recently, Carve et al. (2021) collected EHMC concentrations measured in rivers and lakes globally and calculated risk quotients (RQs) based on the median MEC and a PNEC of 0.4 µg/L. Negligible risk was identified. In addition, the calculated PNEC used an inconsistent assessment factor based on their proposed scheme (i.e., 100 rather than 50), which if amended would have yielded a PNEC of 0.8 µg/L comparable to the PNEC of 1 µg/L derived in the present assessment. Using the revised PNEC, the margin of safety identified would have been even greater, and the RQ for the maximum MEC scenario from the present study would have been less than 1. Three other ERAs were identified (Ma et al., 2016; Tsui et al., 2014; Yan et al., 2018) which all used PNECs derived from the same gene expression endpoint (Zucchi et al., 2011). Gene expression is a nonstandard endpoint in regulatory risk assessment because molecular activity is not directly indicative of adverse effects at the organism or population level (evidenced by the lack of chronic toxicity identified). Thus, these studies were not considered relevant for comparison because they were not in scope for down‐the‐drain risk assessment.

The goal of the present environmental safety assessment was to conduct an ERA for down‐the‐drain releases of EHMC to effluent‐receiving rivers. As such, lakes were beyond the scope of our assessment because they are not representative of the down‐the‐drain emission scenario or the environmental compartment modeled. A different exposure modeling approach would be needed to capture key hydrodynamic properties of lake environments, the emission of UV filters from directly washing off from bathers, and fate processes such as biodegradation for lakes. Monitoring data from lakes were not specifically reviewed, but in river or WWTP monitoring studies which included lakes (Balmer et al., 2005; Cuderman & Heath, 2006; Kameda et al., 2011; Ma et al., 2016; Rodil & Moeder, 2008; Tang et al., 2018), measured concentrations were similar to those observed in rivers (e.g., 90th percentile MEC was 0.02 µg/L).

## CONCLUSION

A freshwater down‐the‐drain risk assessment was conducted for a UV filter of regulatory concern, EHMC, in WWTP effluent‐receiving rivers in the United States. A concentration distribution representative of the spatial variability in population, WWTP location and size, flow, and contribution of EHMC loads from upstream was predicted using the iSTREEM exposure model. When the predicted 90th percentile PEC was compared with the PNEC, risks were negligible. As the level of risk was determined to be negligible, further data collection and refinement of this freshwater EHMC risk assessment are not necessary. To the authors’ knowledge there are currently no freshwater environmental monitoring data available for EHMC in the United States, therefore predicted concentrations were compared with a global distribution of monitoring data relevant to the modeled exposure scenario. Predicted concentrations were substantially higher than measured concentrations, providing confidence in the conservative assumptions applied in the exposure assessment and supporting the use of high‐tier modeling approaches, such as iSTREEM, to conduct robust risk assessments in the absence of refined data (e.g., monitoring data). Ultimately, this innovative approach was leveraged to conduct a novel down‐the‐drain ERA of EHMC emissions across the United States, a prominently affected region not specifically targeted in previous assessments. The present study not only supported current use of EHMC, by demonstrating its ecological safety, but also helps build on an established framework for ERA of high‐interest CPCPs in the United States.

## Supporting Information

The Supporting Information are available on the Wiley Online Library at https://doi.org/10.1002/etc.5488.

## Author Contributions Statement

**Emily E. Burns**: Conceptualization; Methodology; Investigation; Data collection; Formal analysis; Writing—original draft; Writing—review & editing. **Kyle S. Roush**: Conceptualization; Methodology; Investigation; Data collection; Formal analysis; Writing—original draft; Writing—review & editing. **Susan S. Csiszar**: Conceptualization; Methodology; Supervision; Writing—review & editing. **Iain A. Davies**: Conceptualization; Methodology; Supervision; Funding acquisition; Writing—review & editing.

## Supporting information

This article includes online‐only Supporting Information.

Supporting file.

## Data Availability

All data and associated metadata not presented in the Supporting Information are available on request from the corresponding author (daviesi@personalcarecouncil.org). The iSTREEM model is a publicly available resource from the American Cleaning Institute via www.istreem.org.
