# Supplementary material for: Freshwater Environmental Risk Assessment of Down‐the‐Drain Octinoxate Emissions in the United States
Source: Environ Toxicol Chem. 2022 Oct 25;41(12):3116–24. doi: 10.1002/etc.5488 (PMC9828718; doi:10.1002/etc.5488)
Supplement: Supplementary file 1 — Supporting file. [file ETC-41-3116-s001.docx]

Freshwater environmental risk assessment of down-the-drain octinoxate emissions in the United States

Authors: Intentionally blank

Affiliations: Intentionally blank

**SUPPLEMENTARY INFORMATION**

**Figure S1.** Summary of the environmental risk assessment process used in this study. A) grams/per capita per day (g/c/d); B) see Table S2 for iSTREEM input parameters C) see Table 3 for 90^th^ percentile PEC; D) see Table 2 for summary of freshwater toxicity data; E) see Table 2 F) see Table S*3* for assessment factor selection. Predicted environmental concentration (PEC); predicted no-effect concentration (PNEC).

**Table S1**. Summary of ethylhexyl methoxycinnamate (EHMC) physico-chemical properties.

**Table S2.** Summary of the data for the removal of octinoxate (EHMC) in wastewater treatment studies published in the literature. The mean value across the 24 values in this table was used for the iSTREEM modelling. The search was limited to activated sludge treatment as this is the technology utilized in the majority of wastewater treatment plants (WWTPs) in the United States.

**Table S3**. Summary of input to iSTREEM V2.2 to estimate United States mean-flow predicted environmental concentrations (PECs).

**Table S4.** Summary of assessment factors to derive predicted no-effect concentrations (PNECs) in this study. These assessment factors are based on US Environmental Protection Agency guidance (Nabholz 1991; Zeeman and Gilford, 1993; HESI et al. 2022)**.**

**Table S5.** Global monitoring data collected from the literature for EHMC in rivers representative of the down-the-drain release scenario modeled in this study. Studies that were identified as potentially relevant but after detailed assessment were found not to be representative of the down-the-drain release to freshwater scenario modeled in this paper are reported in Table S5. Data presentation varied by study. In Figure 2 (main text) the mean/median was used for distribution calculations; however, when the full data set was provided it was not combined into a single value. When samples were ≤ limit of detection (LOD), the LOD was used in the distribution.

**Table S6.** Environmental monitoring studies of EHMC which were identified as potentially relevant, but after detailed assessment were found to not be representative of the down-the-drain release to freshwater scenario modeled in this paper. Data from these studies was not included in the measured environmental concentration distribution presented in Figure 2 (main text).


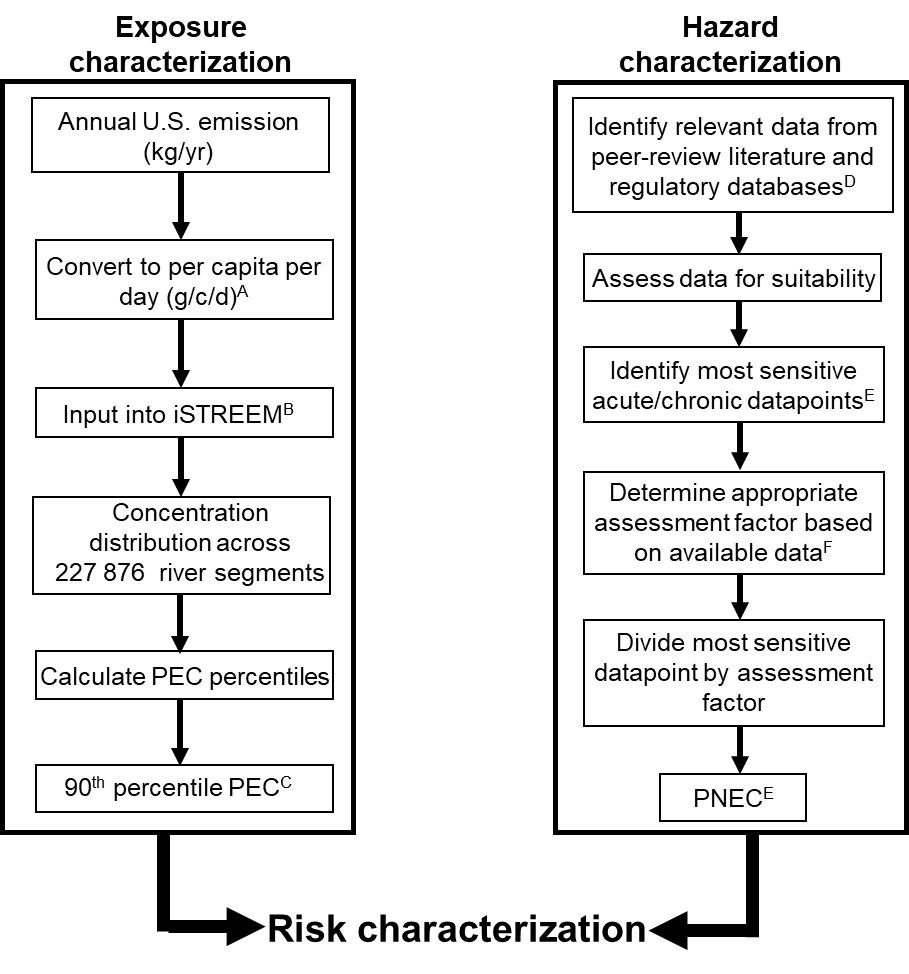


**Figure S1.** Summary of the environmental risk assessment process used in this study. A) grams/per capita per day (g/c/d); B) see Table S2 for iSTREEM input parameters C) see Table 3 for 90^th^ percentile PEC; D) see Table 2 for summary of freshwater toxicity data; E) see Table 2 F) see Table S*3* for assessment factor selection. Predicted environmental concentration (PEC); predicted no-effect concentration (PNEC).

**Table S1**. Summary of ethylhexyl methoxycinnamate (EHMC) physico-chemical properties.

| INCI Name (INN) | CAS RN | Structure | Molecular weight | Solubility^a^ | LogK_ow_^a^ |
| --- | --- | --- | --- | --- | --- |
| Ethylhexyl methoxycinnamate (Octinoxate) | 5466-77-3; 83834-59-7 | 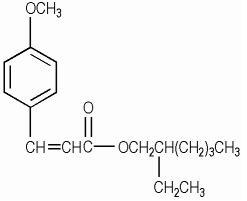 | 290.4 | 51 µg/L | 6 |
| ^a^ECHA (2022) Registration Dossier  CAS RN = Chemical Abstracts Service Registry Number INCI = International Nomenclature of Cosmetic Ingredients; INN = International nonproprietary name; Kow = octanol-water partition coefficient. | | | | | |

**Table S2.** Summary of the data for the removal of octinoxate (EHMC) in wastewater treatment studies published in the literature. The mean value across the 24 values in this table was used for the iSTREEM modelling. The search was limited to activated sludge treatment as this is the technology utilized in the majority of wastewater treatment plants (WWTPs) in the United States.

| **Mean removal (standard deviation)** | **Mean removal value(s) or range reported** | **Reference** |
| --- | --- | --- |
| 83.5% (18.8) | 97%, 97%, 99%, 99%, 98%, 99%, 99%, 98%, 99% | Balmer et al. (2005) |
|  | 99% (April), 84% (October) | Liu et al. (2012)^1,2^ |
|  | 84% | Bueno et al. (2012)^1^ |
|  | 84% | Ekpeghere et al. (2016)^1^ |
|  | 99% | Tsui et al. (2014a)^3^ |
|  | 90% | Gackowska (2020) |
|  | 99% | Li et al. (2022)^1^ |
|  | 70%, 71%, 50%, 50%, 63%, 39%, 67%, 72% | Juksu et al. (2020) |

^1^ Values estimated from a graph using WebPlot Digitizer (Rohatgi 2020).

^2^ Removal values calculated by dividing the secondary effluent concentration (activated sludge) by the initial influent concentration. These removals were added together for consistency with other reported values.

^3^Reports in the text that EHMC was 99% removed in Plant E, the only activated sludge WWTP studied.

**Table S3**. Summary of input to iSTREEM V2.2 to estimate United States mean-flow predicted environmental concentrations (PECs).

| **Parameter** | **Input** |
| --- | --- |
| WWTP removal for: Activated sludge, oxidation ditch, lagoon, trickling filter, and rotating biological contractor | 83.5% |
| Primary removal | 0% |
| In-stream decay (d^-1^) | 0.047 d^-1^ |
| Emission | 0.0086 g/c/d |

**Table S4.** Summary of assessment factors to derive predicted no-effect concentrations (PNECs) in this study. These assessment factors are based on US Environmental Protection Agency guidance (Nabholz 1991; Zeeman and Gilford, 1993; HESI et al. 2022)**.**

| **Toxicity data available** | **Assessment Factor** | |
| --- | --- | --- |
| Predicted (QSAR) | 1000 | |
| Acute data (1 or 2 taxa) | 1000 | |
| Acute data (3 taxa) | 100 | |
| Chronic data^1^ (1 or more taxa) | 10 | |
| Field study (e.g. microcosm/mesocosm) | 1 | |
| ^1^To apply the chronic data assessment factor (i.e. 10), it must be applied to the most sensitive taxa. The acute toxicity profile can be useful for this purpose. | |  |

**Table S5.** Global monitoring data collected from the literature for EHMC in rivers representative of the down-the-drain release scenario modeled in this study. Studies that were identified as potentially relevant but after detailed assessment were found not to be representative of the down-the-drain release to freshwater scenario modeled in this paper are reported in Table S5. Data presentation varied by study. In Figure 2 (main text) the mean/median was used for distribution calculations; however, when the full data set was provided it was not combined into a single value. When samples were ≤ limit of detection (LOD), the LOD was used in the distribution.

| **Country** | **Mean and/or *median* (ng/L)** | **Samples, detection frequency** | **Individual data points?** | **Description** | **Reference** |
| --- | --- | --- | --- | --- | --- |
| Germany | 21 (River Elsterbecken) <LOD (River Parthe) | 4, not reported | No, average | Rivers and lakes studied. The lakes were selected to cover inputs from recreational inputs, while the rivers receive WWTP effluents from Leipzig. Samples collected in May 2007. LOD/LOQ: 16/26 ng/L. | Rodil and Moeder (2008) |
| Germany | N.D. | 23, 0% |  | Samples were collected along beaches and in estuaries of the Baltic Sea. Samples were also collected further up river which would be suitable for consideration in this study. A total of 23 samples were collected in July 2015 that were identified as representative of the modelled exposure scenario. LOD: 5 ng/L. | Fisch et al. (2017) |
| Italy | N.D. | 11, 9% |  | Eleven samples were collected from the Po River in February 2020. LOD/LOQ: 0.9/3.0 ng/L. | Riboni et al. (2021) |
| Ireland | 3.8, *2.6* (rural river)  1.1, *0.8* (urban river) | 12, 75%  12, 17% | Yes | A rural and urban sampling site were visited monthly for a year. The river was sampled 50 m downstream in the rural location and 1 km downstream from a WWTP in the urban location. LOD/LOQ: 1.2/3.65 ng/L. | Wright (2021) |
| Norway | 0.12 (June)  0.75 (September) | 3, 100%  3, 100% | No | Grab samples were collected from a single site in the River Alna, Norway. Samples were collected on three different dates per month and the average reported. The LOD and LOQ were not reported, but there was 100% detection frequency. | (Allan et al. 2022) |
| Portugal | 44.9, *29.4* (Leça River) 40, 9.0 (Antuã River) 77.6, *30.0* (Cértima River) | 14, 79%  18, 61%  16,75% | Yes | Sampling was conducted once in the wet and dry seasons in four different rivers: Ave, Leça, Antuã, and Cértima. There were 8 – 9 sampling points along each river that were selected based on various sources such as WWTPs, agriculture, industrial areas and residential areas. It is stated the water quality in the River Ave is heavily affected by industries including: textiles, rubber manufacture, leather tanning, and plastic production. Therefore, the data from the Ave River was not included. LOD/LOQ: 1.52/9.58 ng/L. | Barbosa et al. (2018) |
| **Table S5.** Continued. | | | | | |
| Country | Mean and/or *median* (ng/L) | Samples, detection frequency | Individual data points? | Description | Reference |
| Portugal | N.D. (Leça)  N.D. (Antuã)  N.D. *(*Cértima) | 16, 13%  18, 28%  16, 19% |  | Samples were collected from the Ave, Leça, Antuã, and Cértima rivers in summer and winter 2016/2017. Similar sites were visited in comparison to Barbosa et al. (2018). The Ave River was not included due industrial sources. The LOD/LOQ was 0.14/0.48 ng/L. | Homem et al. (2022) |
| Switzerland | 6 | 2, 100% | No | Single sample site in the River Glatt, samples collected twice. The goal of collecting the grab samples was to calibrate POCIS passive samplers (data not included). September and October 2007. The LOD was not reported. | Fent et al. (2010) |
| Switzerland | 5.5 | 47, not reported | No, average | Unpublished data from a Swiss Water Protection Board water monitoring station on the River Rhine, downstream of Basel at the Palmrain bridge. Collected in the summer of 1997. The LOD is assumed to be 2 ng/L, consistent with similar studies cited by and used by the author. | Straub (2002) |
| Australia | 0.169, *0.014* | 4, 100% | Yes | Samples were collected from four different rivers that flow into Port Philip Bay, Victoria. | Allinson et al. (2018) |
| China | 13.2, *16.9* (North river) 9.6, *5.3* (West river) | 26, 77%  44, 100% | Yes | Samples collected from two rivers that are major drinking water sources in the Pearl River. There were 22 sampling sites in the West river and 13 in the North river. Samples were collected once during the wet and dry seasons. The study states that the UV filter concentrations observed were mainly from wastewater effluent, runoff, and domestic discharge. LOD/LOQ: 0.03/0.11 ng/L. | Hu et al. (2021) |
| China | 1.3, *0.84*  *1.0* (warm season)  *<LOD* (cold season) | 96, 81% | No, only median from each of the two sampling campaigns | Samples were collected in northeast China from the Songhua River, second Songhua River and Nen River. There were 27 sampling sites and two sampling campaigns June – July and September – October 2017. The median for each season is reported. LOD: 0.74 ng/L. | Li et al. (2021) |

| **Table S5.** Continued. | | | | | |
| --- | --- | --- | --- | --- | --- |
| Country | Mean and/or *median* (ng/L) | Samples, detection frequency | Individual data points? | Description | Reference |
| China | *2.2* | 57, 96% | No, only the median is reported | This study takes place in two rivers in the Jiulong River Basin, north of the Pearl River. There are 11 sampling sites in the norther river and 8 in the western river. The estuary downstream was also studied, but this data was not included as it is not representative of the down-the-drain freshwater UV filter discharge scenario. Samples were collected three times, in January, June, and September 2013. LOD: 0.01 ng/L. | Lv et al. (2014) |
| China | 2.3, *2.2* | 8, 63% | Yes | River samples were collected in tributaries of the Yangzte River in the Nanjing region. Lakes with significant recreational influence were also studied. The Xuanwu lake drains into the Jinchuan River and therefore samples from this river are not representative of the down-the-drain release scenario. Only sites R1 – R5, R8, R9, and R11 were appropriate to include as the other sites in the study had recreational inputs. LOD: 0.65 ng/L. | Ma et al. (2016) |
| China | 32.3 (Tangxi River)  12.7 (Nanfei River)  28.1 (Banqiao River)  6.09 (Dianbu River)  8.96 (Paihe River) | 2, not reported  10, not reported  5, not reported 5, not reported 2, not reported | No, just mean per river | A total of 24 samples were collected from five rivers which flow into Lake Chaohu. Samples were collected in 2017. Samples were also collected from the lake, but these data were not included as they are not representative of the modeled scenario. LOQ: 0.47 ng/L. | Tang et al. (2018) |
| China | 0.008, *0.008* | 9, 100% | Yes | Samples were collected in triplicate at 10 sites along the Yangtze River. Only sites S1 – S9 were included to avoid interference from the South China Sea. | (Yan et al. 2018) |
| Japan | 26 | 12, 25% | No, average | Collected samples from rivers in the Saitama Prefecture in September and August 2008. Five scenarios were studied: 1) Two streams with direct inputs of untreated wastewater, 2) WWTP effluents 3) six rivers heavily polluted by industrial and domestic wastewaters and 4) moderately contaminated rivers (WWTPs), 5) background samples were also collected but they are impacted by recreational use. Only scenario 4 is included as the other scenarios are not representative of the modeled scenario. LOD: 0.1 ng/L. | Kameda et al. (2011) |

| **Table S5.** Continued. | | | | | |
| --- | --- | --- | --- | --- | --- |
| Country | Mean and/or *median* (ng/L) | Samples, detection frequency | Individual data points? | Description | Reference |
| Thailand | *101* | 80, not reported. | No, median estimated from box plot | Samples were collected in Bangkok and Pattaya. In addition to the river data, samples were also collected from WWTPs, biota, sediment, and the marine environment. Only the river data is included. LOD/LOQ: 2.39/7.96 ng/L. | Juksu et al. (2020) |
| Thailand | 91.5, *91.5* | 2, 100% | Yes | River receiving municipal wastewater was sampled in Bangkok at the Saen Saep Canal and the Chao Phraya River in August 2013. No other samples collected in this study were appropriate as they were from the marine environment. LOD: 0.1 ng/L. | Tsui et al. (2014b) |
| South Korea | *52* | 33, > 90% | No, presented as box plot | Samples were collected from 11 sites along three rivers, Nakdong River, Guemho River, and Nam River. Samples were collected in spring, summer, and fall. Median estimated from graph. LOD/LOQ: 1.43/4.75 ng/L. | Ekpeghere et al. (2016) |
| South Korea | 58.3, *47* (Suyeong River)  74.6, *66* (Songjeong Stream) | 3, 100%  3, 100% | Yes | Samples were collected in two rivers, Songjeong Stream and Suyeong River on three occasions (July, August, September 2013). Samples were also collected from beaches and WWTPs, but these were not included. Concentrations estimated from graph. LOD/LOQ: 2.1/6.9 ng/L. | Kim et al. (2017) |
|  | | | | | |
|  |  |  |  |  |  |

**Table S5.** Environmental monitoring studies of EHMC which were identified, but after detailed assessment were found to not be representative of the down-the-drain release to freshwater scenario modeled in this paper. Data from these studies was not included in the measured environmental concentration distribution presented in Figure 2 (main text).

| **Region** | **Country** | **Description** | **Reason for rejection** | **Reference** |
| --- | --- | --- | --- | --- |
| Asia | China | Drinking water reservoirs were sampled at 12 sites. The Jiangang, Baiguishan, and Nanwan Reservoir and the Shahe Water Source Area. | Reservoirs rather than rivers were sampled. This is not representative of the modeled United States scenario (down-the-drain release) | Wu et al. (2019) |
| Europe | Romania | Three main rivers in central Transylvania were sampling in 9 locations during two sampling campaigns. Only the mean of the 18 samples was reported. | It cannot be 100% determined which study compound corresponds to octinoxate due to the spelling and use of acronyms. For example, 2-Ethylhexyl-4-metoxycinnamate has acronym PRM and 2-Ethyltrans-4-metoxycinnamate has acronym EHMC. | Feher et al. (2016) |
| Europe | Italy | Samples were collected once monthly from April to August at the mouth of the river Sturla in Genoa. | The mouth of the river Sturla is on the Mediterranean Sea and is between two recreational beaches. Therefore, there is the potential for contributions of octinoxate from recreational use which would not be representative of the modeled scenario. | Magi et al. (2012) |
| Europe | Slovenia | Samples were collected from the River Nadiža, River Kokra, and the River Kolpa in August 2004. Lakes, swimming pools, beaches, wastewater influent and effluent were also sampled. | The goal of the study was to sample recreational waters during high use season to determine the UV filter input from this source. This was paired with WWTP samples to understand the input from recreation and down-the-drain. Therefore, the river samples collected in this study are not representative of the down-the-drain release scenario modeled in the study. | Cuderman and Heath (2006) |
| South America | Brazil | Samples were collected in the Araraquara region of Brazil at three locations monthly. Samples were drinking water plant influent. | It is implied that this is river water, but it is not clear what the sources of UV filters to these rivers are. Further, there are very few detections in the dataset, only 10%. This is likely because the LOQ and the LOD are quite high compared to other studies, 78.2 and 23.5 ng/L, respectively. This means 90% of the dataset would need to be censored and the high LOQ and LOD could artificially inflate concentrations. Therefore, due to the unclear source of the drinking water treatment plant influent, limited detections and high LOQ/LOD, and noted sampled contamination this study was discarded. | da Silva et al. (2015) |

| **Table S5**. Continued. | | | | |
| --- | --- | --- | --- | --- |
| Region | Country | Description | Reason for rejection | Reference |
| Europe | Portugal | A temporal monitoring campaign in two highly polluted river in Northern Portugal (the Ave and Sousa rivers). Sampling sites were chosen based on the locations of tributaries, WWTPs and industry. There were 15 sampling points per river which were visited in May, August, November, and February. | The rivers are impacted by the industry in the region which includes textile, rubber manufacturing, leather tanning, plastic production, and furniture production. There is also incidence of known illegal discharges from these industries into the Ave river. Information on the industries impacting the Sousa river were discussed by Teixeira (2020). The seasonal data indicates the industries are likely a source of octinoxate to the river. Due to the industrial influence on octinoxate loading in the rivers, this dataset was not selected as it is not representative of the down-the-drain release scenario. | Sousa et al. (2019) |

**REFERENCES**

Allan I, Jenssen MTS, Baek K, Kaste O. 2022. The Norwegian River Monitoring Programme: Priority substances and emerging contaminants in selected Norwegian rivers. Report No. 7688-2022.

Allinson M, Kameda Y, Kimura K, Allinson G. 2018. Occurrence and assessment of the risk of ultraviolet filters and light stabilizers in Victorian estuaries. Environ Sci Pollut Res. 25(12):12022–12033. doi:10.1007/s11356-018-1386-7.

Balmer ME, Buser HR, Müller MD, Poiger T. 2005. Occurrence of some organic UV filters in wastewater, in surface waters, and in fish from Swiss lakes. Environ Sci Technol. 39:953–962. doi:10.1021/es040055r.

Barbosa MO, Ribeiro AR, Ratola N, Hain E, Homem V, Pereira MFR, Blaney L, Silva AMT. 2018. Spatial and seasonal occurrence of micropollutants in four Portuguese rivers and a case study for fluorescence excitation-emission matrices. Sci Total Environ. 644:1128–1140. doi:10.1016/j.scitotenv.2018.06.355. https://doi.org/10.1016/j.scitotenv.2018.06.355.

Bueno MJM, Gomez MJ, Herrera S, Hernando MD, Agüera A, Fernández-Alba AR. 2012. Occurrence and persistence of organic emerging contaminants and priority pollutants in five sewage treatment plants of Spain: Two years pilot survey monitoring. Environ Pollut. 164:267–273. doi:10.1016/j.envpol.2012.01.038. http://dx.doi.org/10.1016/j.envpol.2012.01.038.

Cuderman P, Heath E. 2006. Determination of UV filters and antimicrobial agents in environmental water samples. Anal Bioanal Chem. 387:1343–1350. doi:10.1007/S00216-006-0927-Y.

Ekpeghere KI, Kim UJ, O SH, Kim HY, Oh JE. 2016. Distribution and seasonal occurrence of UV filters in rivers and wastewater treatment plants in Korea. Sci Total Environ. 542:121–128. doi:10.1016/j.scitotenv.2015.10.033. http://dx.doi.org/10.1016/j.scitotenv.2015.10.033.

Fent K, Zenker A, Rapp M. 2010. Widespread occurrence of estrogenic UV-filters in aquatic ecosystems in Switzerland. Environ Pollut. 158(5):1817–1824. doi:10.1016/j.envpol.2009.11.005. http://dx.doi.org/10.1016/j.envpol.2009.11.005.

Feher IC, Moldovan Z, Oprean I. 2016. Spatial and seasonal variation of organic pollutants in surface water using multivariate statistical techniques. Water Sci Technol. 74(7):1726–1735. doi:10.2166/WST.2016.351.

Fisch K, Waniek JJ, Schulz-Bull DE. 2017. Occurrence of pharmaceuticals and UV-filters in riverine run-offs and waters of the German Baltic Sea. Mar Pollut Bull. 124(1):388–399. doi:10.1016/j.marpolbul.2017.07.057. http://dx.doi.org/10.1016/j.marpolbul.2017.07.057.

Gackowska A, Studziński W. 2020. Effect of Activated Sludge on the Degradation of 2-Ethylhexyl 4-Methoxycinnamate and 2-Ethylhexyl 4-(Dimethylamino)Benzoate in Wastewater. Water Air Soil Pollut. 231(158).

Health and Environmental Sciences Institute (HESI). 2022. EnviroTox Database & Tools. Version 2.0.0 Available: http://www.envirotoxdatabase.org/ (Retrieved 4/7/2022)

Homem V, Llompart M, Vila M, Ribeiro ARL, Garcia-Jares C, Ratola N, Celeiro M. 2022. Gone with the flow - Assessment of personal care products in Portuguese rivers. Chemosphere.:133552. doi:10.1016/j.chemosphere.2022.133552. https://doi.org/10.1016/j.chemosphere.2022.133552.

Hu LX, Cheng YX, Wu D, Fan L, Zhao JH, Xiong Q, Chen Q Le, Liu YS, Ying GG. 2021. Continuous input of organic ultraviolet filters and benzothiazoles threatens the surface water and sediment of two major rivers in the Pearl River Basin. Sci Total Environ. 798:149299. doi:10.1016/j.scitotenv.2021.149299. https://doi.org/10.1016/j.scitotenv.2021.149299.

Juksu K, Liu Y-S, Zhao J-L, Yao L, Sarin C, Sreesai S, Klomjek P, Traitangwong A, Ying G-G. 2020. Emerging contaminants in aquatic environments and coastal waters affected by urban wastewater discharge in Thailand: An ecological risk pespective. Ecotoxicol Environ Saf. 204:110952. doi:https://doi.org/10.1016/j.ecoenv.2020.110952.

Kameda Y, Kimura K, Miyazaki M. 2011. Occurrence and profiles of organic sun-blocking agents in surface waters and sediments in Japanese rivers and lakes. Environ Pollut. 159(6):1570–1576. doi:10.1016/j.envpol.2011.02.055. http://dx.doi.org/10.1016/j.envpol.2011.02.055.

Kim KY, Ekpeghere KI, Jeong HJ, Oh JE. 2017. Effects of the summer holiday season on UV filter and illicit drug concentrations in the Korean wastewater system and aquatic environment. Environ Pollut. 227:587–595. doi:10.1016/J.ENVPOL.2017.04.055.

Li W-L, Zhang Z-F, Kilgallon J, Sparham C, Li Y-F, Yuan Y-X. 2022. Fate of household and personal care chemicals in typical urban wastewater treatment plants indicate different seasonal patterns and removal mechanisms. Environ Pollut. 294(December 2021):118618. doi:10.1016/j.envpol.2021.118618. https://doi.org/10.1016/j.envpol.2021.118618.

Li WL, Zhang ZF, Li YF, Hung H, Yuan YX. 2021. Assessing the distributions and fate of household and personal care chemicals (HPCCs) in the Songhua Catchment, Northeast China. Sci Total Environ. 786:147484. doi:10.1016/j.scitotenv.2021.147484. https://doi.org/10.1016/j.scitotenv.2021.147484.

Liu YS, Ying GG, Shareef A, Kookana RS. 2012. Occurrence and removal of benzotriazoles and ultraviolet filters in a municipal wastewater treatment plant. Environ Pollut. 165:225–232. doi:10.1016/j.envpol.2011.10.009. http://dx.doi.org/10.1016/j.envpol.2011.10.009.

Lv M, Sun Q, Hu A, Hou L, Li J, Cai X, Yu CP. 2014. Pharmaceuticals and personal care products in a mesoscale subtropical watershed and their application as sewage markers. J Hazard Mater. 280:696–705. doi:10.1016/j.jhazmat.2014.08.054. http://dx.doi.org/10.1016/j.jhazmat.2014.08.054.

Ma B, Lu G, Liu F, Nie Y, Zhang Z, Li Y. 2016. Organic UV Filters in the Surface Water of Nanjing, China: Occurrence, Distribution and Ecological Risk Assessment. Bull Environ Contam Toxicol. 96(4):530–535. doi:10.1007/s00128-015-1725-z.

Magi E, Di Carro M, Scapolla C, Nguyen KTN. 2012. Stir Bar Sorptive Extraction and LC–MS/MS for Trace Analysis of UV Filters in Different Water Matrices. Chromatogr. 75(17):973–982. doi:10.1007/S10337-012-2202-Z.

Nabholz J V. 1991. Environmental hazard and risk assessment under the United States Toxic Substances Control Act. Sci Total Environ. 109–110:649–665. doi:doi.org/10.1016/0048-9697(91)90218-4.

Riboni N, Fornari F, Bianchi F, Careri M. 2021. A simple and efficient Solid-Phase Microextraction – Gas Chromatography – Mass Spectrometry method for the determination of fragrance materials at ultra-trace levels in water samples using multi-walled carbon nanotubes as innovative coating. Talanta. 224:121891. doi:10.1016/J.TALANTA.2020.121891.

Rodil R, Moeder M. 2008. Development of a method for the determination of UV filters in water samples using stir bar sorptive extraction and thermal desorption-gas chromatography-mass spectrometry. J Chromatogr A. 1179(2):81–88. doi:10.1016/j.chroma.2007.11.090.

Rohatgi A. 2020. WebPlotDigitizer, Version 4.3. Pacifica, CA, USA. [accessed April. 7, 2022]. https://automeris.io/WebPlotDigitizer

da Silva CP, Emídio ES, de Marchi MRR. 2015. The occurrence of UV filters in natural and drinking water in São Paulo State (Brazil). Environ Sci Pollut Res. 22(24):19706–19715. doi:10.1007/s11356-015-5174-3.

Sousa JCG, Ribeiro AR, Barbosa MO, Ribeiro C, Tiritan ME, Pereira MFR, Silva AMT. 2019. Monitoring of the 17 EU Watch List contaminants of emerging concern in the Ave and the Sousa Rivers. Sci Total Environ. 649:1083–1095. doi:10.1016/j.scitotenv.2018.08.309.

Straub JO. 2002. Concentrations of the UV filter ethylhexyl methoxycinnamate in the aquatic compartment: a comparison of modelled concentrations for Swiss surface waters with empirical monitoring data. Toxicol Lett. 131(1–2):29–37. doi:10.1016/S0378-4274(02)00042-5.

Tang Z, Han X, Li G, Tian S, Yang Y, Zhong F, Han Y, Yang J. 2018. Occurrence, distribution and ecological risk of ultraviolet absorbents in water and sediment from Lake Chaohu and its inflowing rivers, China. Ecotoxicol Environ Saf. 164(May):540–547. doi:10.1016/j.ecoenv.2018.08.045. https://doi.org/10.1016/j.ecoenv.2018.08.045.

Teixeira MJN. 2020. Biomonitoring of Ferreira and Sousa rivers using diatoms: comparison between morphological and molecular approaches. Universidade de Aveiro.

Tsui MMP, Leung HW, Lam PKS, Murphy MB. 2014a. Seasonal occurrence, removal efficiencies and preliminary risk assessment of multiple classes of organic UV filters in wastewater treatment plants. Water Res. 53:58–67. doi:10.1016/j.watres.2014.01.014.

Tsui MMP, Leung HW, Wai TC, Yamashita N, Taniyasu S, Liu W, Lam PKS, Murphy MB. 2014b. Occurrence, distribution and ecological risk assessment of multiple classes of UV filters in surface waters from different countries. Water Res. 67:55–65. doi:10.1016/j.watres.2014.09.013. http://dx.doi.org/10.1016/j.watres.2014.09.013.

Wright HR. 2021. Novel detection and risk assessment of contaminants of emerging concern in a range of aquatic matrices in Ireland. Dublin City University.

Wu D, Zhou Y, Lu G, Hu K, Yao J, Shen X, Wei L. 2019. The occurrence and risks of selected emerging pollutants in drinking water source areas in Henan, China. Int J Environ Res Public Health. 16(21). doi:10.3390/ijerph16214109.

Yan Z, Yang H, Dong H, Ma B, Sun H, Pan T, Jiang R, Zhou R, Shen J, Liu J, et al. 2018. Occurrence and ecological risk assessment of organic micropollutants in the lower reaches of the Yangtze River, China: A case study of water diversion. Environ Pollut. 239:223–232. doi:10.1016/j.envpol.2018.04.023. https://doi.org/10.1016/j.envpol.2018.04.023.

Zeeman, M., & Gilford, J. (1993). Ecological hazard evaluation and risk assessment under EPA's Toxic Substances Control Act (TSCA): An introduction. In W. G. Landis, J. S. Hughes, & M. A. Lewis (Eds.), Environmental toxicology and risk assessment, ASTM STP 1179 (pp. 7–21). American Society for Testing and Materials. https://doi.org/10.1520/ STP19230S
